# Draft genome sequence of potential probiotic *Lacticaseibacillus paracasei* subsp. *paracasei* S1 isolated from *Nypa fruticans* Wurmb. in Cagayan, Northern Philippines

**DOI:** 10.1128/mra.01227-25

**Published:** 2026-03-16

**Authors:** Lemuel Ray Balloyan, Jimmbeth Zenila Fabia, Ma. Joy Theresa Agcaoili, James Patrick Acang, James Patrick Peralta, Peter James Icalia-Gann

**Affiliations:** 1 Center for Cellular and Molecular Medical Research, College of Medicine, Mariano Marcos State University 69374, Batac, Ilocos Norte, Philippines; 2 Research Directorate, Mariano Marcos State University 69374, Batac, Ilocos Norte, Philippines; 3 Computer Science Department, Mariano Marcos State University 69374, Batac, Ilocos Norte, Philippines; 4 Department of Medical Microbiology, College of Medicine, Mariano Marcos State University 69374, Batac, Ilocos Norte, Philippines; 5 Department of Biochemistry and Molecular Biology, College of Medicine, Mariano Marcos State University 69374, Batac, Ilocos Norte, Philippines; University of Wisconsin-Madison, Madison, Wisconsin, USA

**Keywords:** whole-genome sequence

## Abstract

Here, we present the draft genome assembly of a putative probiotic *Lactocaseibacillus paracasei* subsp. *paracasei* strain S1 isolated from *Nypa fruticans*. The *L. paracasei* subsp. *paracasei* strain S1 genome is 3.1 Mbp in size, and its annotation revealed the presence of genes involved in several probiotic functionalities.

## ANNOUNCEMENT

Due to numerous health benefits, probiotics have emerged as the most prominent ingredient in the functional food era (1). Here, we used whole-genome sequencing to establish the functionality and safety of our potential probiotic *L. paracasei* subsp. *paracasei* S1 for food and pharmaceutical use. Strain S1 was isolated from *Nypa fruticans* collected from Pamplona, Cagayan (1,827.8470 N°, 12,120.4333 E°). The naturally fermenting sap of the plant was directly streaked onto de Man, Rogosa and Sharpe (MRS) agar. Plates were incubated at 37°C, and colony growth was observed after 24 h. Colonies exhibiting lactic acid bacteria morphology were subjected to subsequent rounds of purification using the same media and growth conditions. Pure cultures of S1 were preserved in 50% glycerol (v/v), stored in −81°C freezer, and are being maintained at the Center for Cellular and Molecular Medical Research in Batac, Ilocos Norte.

The *L. paracasei* subsp. *paracasei* strain S1 was cultured in MRS broth and incubated for 24 h prior to DNA extraction. Approximately 1 mL from the broth culture was aliquoted and centrifuged following the protocols of the GF-1 Bacterial DNA Extraction Kit (Vivantis Technologies, Malaysia). The concentration and quality of the extracted DNA were assessed visually using agarose gel electrophoresis (AGE) and quantified using the spectrophotometry-based ScanDrop^2^ (Analytik Jena, Germany). High-quality DNA was then sent to the DNA Sequencing Core Facility, Philippine Genome Center, Philippines. A DNA library was prepared using the Nextera XT DNA Library Preparation Kit (Illumina Inc., USA) and quality checked using Agilent TapeStation 2200 (Agilent Technologies, USA). Whole-genome sequencing was performed using Illumina MiSeq (Illumina, Inc., USA).

Prior to assembly, the quality of the raw reads was assessed using FastQC v0.12.1 (2). A total of 1,124,122 raw reads were generated with read length ranging from 35 to 151 bp and an expected coverage of 55×. Trimmomatic v0.40 (3) was used to remove adapters and low-quality bases with a quality score cutoff ≥ Q20 and minimum length of >36 base pairs. *De novo* assembly of the genome was conducted using SPAdes v4.2.0 (4). Genome completeness and the quality of the assembled genome were assessed using QUAST v5.3.0, BUSCO v6.0.0, and checkM2 v1.1.0 (5–7). The annotation tool Bakta v1.11.3 (8) was then used to annotate the genome with the resulting annotation viewed in the web-based tool Bakta Viewer. Default parameters for the different software were used, except where otherwise noted.

In this study, the draft assembly of strain S1 generated a genome size of 3.1 Mbp with 99% BUSCO score. The genome assembly and annotation results are summarized in Table 1. Submitting the draft genome into the MiGA webserver (9) reveals the top result being *Lactocaseibacillus paracasei* subsp. *paracasei* GCF 004354655 1 (ATCC 25302) having a 98.3% average nucleotide identity (ANI). The presence of genes like *fliK* (10) and *dlt* operon (11) for adhesion, *cps* gene cluster (12) for immunomodulation, *nagB* (13) and *pyrG* for bile tolerance, and BFOOKN_00465 for bacteriocin production was found in the genome. The annotated draft assembly of strain S1 generated in this study will support further studies on its potential for functional food and pharmaceutical use.

**TABLE 1:** Assembly and annotation results of strain S1

Assembly statistics	S1
Number of contigs	41
Genome size (bp)	3,060,919
Largest contig (bp)	651,286
GC content (%)	46.12
N50	350,017
L50	3
Coding density (%)	85%
CheckM2 completeness	99.94%
BUSCO (lactobacillaceae_odb12)	99%
# of proteins	2,867
tRNA	57
rRNA	3

## Data Availability

The whole genome data of *Lacticaseibacillus paracasei* subsp. *paracasei* S1 can be obtained under accession JBTNPG000000000.1 in GenBank. The BioSample and BioProject accession numbers are SAMN50018499 and PRJNA1293312, respectively. The raw sequences can be accessed in NCBI with SRA accession SRR35844808.
